# The haloarchaeal MCM proteins: bioinformatic analysis and targeted mutagenesis of the β7-β8 and β9-β10 hairpin loops and conserved zinc binding domain cysteines

**DOI:** 10.3389/fmicb.2014.00123

**Published:** 2014-03-26

**Authors:** Tatjana P. Kristensen, Reeja Maria Cherian, Fiona C. Gray, Stuart A. MacNeill

**Affiliations:** ^1^Department of Biology, University of Copenhagen, Københavns BiocenterCopenhagen N, Denmark; ^2^School of Biology, University of St. AndrewsNorth Haugh, St. Andrews, Fife, UK

**Keywords:** *Haloferax volcanii*, archaea, Haloarchaea, MCM helicase, DNA replication, reverse genetics, zinc binding domain

## Abstract

The hexameric MCM complex is the catalytic core of the replicative helicase in eukaryotic and archaeal cells. Here we describe the first *in vivo* analysis of archaeal MCM protein structure and function relationships using the genetically tractable haloarchaeon *Haloferax volcanii* as a model system. *Hfx. volcanii* encodes a single MCM protein that is part of the previously identified core group of haloarchaeal MCM proteins. Three structural features of the N-terminal domain of the *Hfx. volcanii* MCM protein were targeted for mutagenesis: the β7-β8 and β9-β10 β-hairpin loops and putative zinc binding domain. Five strains carrying single point mutations in the β7-β8 β-hairpin loop were constructed, none of which displayed impaired cell growth under normal conditions or when treated with the DNA damaging agent mitomycin C. However, short sequence deletions within the β7-β8 β-hairpin were not tolerated and neither was replacement of the highly conserved residue glutamate 187 with alanine. Six strains carrying paired alanine substitutions within the β9-β10 β-hairpin loop were constructed, leading to the conclusion that no individual amino acid within that hairpin loop is absolutely required for MCM function, although one of the mutant strains displays greatly enhanced sensitivity to mitomycin C. Deletions of two or four amino acids from the β9-β10 β-hairpin were tolerated but mutants carrying larger deletions were inviable. Similarly, it was not possible to construct mutants in which any of the conserved zinc binding cysteines was replaced with alanine, underlining the likely importance of zinc binding for MCM function. The results of these studies demonstrate the feasibility of using *Hfx. volcanii* as a model system for reverse genetic analysis of archaeal MCM protein function and provide important confirmation of the *in vivo* importance of conserved structural features identified by previous bioinformatic, biochemical and structural studies.

## Introduction

In all forms of life, successful chromosomal DNA replication requires efficient unwinding of the DNA double helix at the replication forks, a reaction catalyzed by the replicative DNA helicase. In eukaryotes the replicative helicase is the CMG complex, a tripartite molecular machine composed of Cdc45, MCM and GINS (reviewed by Onesti and MacNeill, 2013). The MCM (mini-chromosome maintenance) complex is the catalytic core of this machine (Vijayraghavan and Schwacha, 2012). MCM is a ring-shaped hexamer composed of six related but non-identical subunits, each of which is a member of the AAA+ (ATPases associated with diverse cellular activities) protein superfamily (Duderstadt and Berger, 2008). MCM is loaded onto chromosomal replication origins as a head-to-head double hexamer in the G1 phase of the cell cycle in a reaction known as replication licensing (Evrin et al., 2009; Remus et al., 2009; Gambus et al., 2011). Cdc45 and GINS then assemble at the G1-S boundary to form the CMG, activation of which involves MCM subunit phosphorylation by DDK (Dbf4-dependent protein kinase). Once activated, individual CMG complexes move with the replication forks from origin to inter-origin sequences.

Consistent with their shared evolutionary history, homologs of the major eukaryotic replication factors have been identified and characterized in the archaea, including homologs of the three components of the CMG. Owing to their high sequence similarity to their eukaryotic counterparts, archaeal MCM proteins were the first to be identified and biochemically characterized. Many archaea encode single MCM proteins that have been shown to form—or are presumed to form—homohexameric helicase complexes (reviewed by Slaymaker and Chen, 2012). The best studied examples of this type of MCM are from the euryarchaeon *Methanothermobacter thermoautotrophicum* and the crenarchaeon *Sulfolobus solfataricus*. However, a number of species encode multiple MCM proteins, such as *Thermococcus kodakarensis* and *Methanococcus maripaludis*, which encode three and four, respectively (Walters and Chong, 2010; Ishino et al., 2011; Pan et al., 2011). It is not impossible that in some species these proteins could form eukaryotic-like heterohexameric complexes *in vivo*. Interestingly, only one of the three *T. kodakarensis* MCM proteins is essential for cell viability (Ishino et al., 2011; Pan et al., 2011)

Structural information is available for a number of archaeal MCM proteins and has been used to guide biochemical investigations of protein structure-function relationships (reviewed by Slaymaker and Chen, 2012). At a structural level, individual archaeal MCM proteins comprise a non-catalytic N-terminal domain followed by the catalytic AAA+ domain and, at the extreme C-terminus, a short winged helix-turned-helix (wHTH) domain. The most extensive crystal structure is that of near full-length *S. solfataricus* MCM which spans the N-terminal and catalytic AAA+ domains but not the wHTH domain (Brewster et al., 2008). Efforts to determine the structure of the wHTH by NMR are ongoing (Wiedemann et al., 2013). Additional crystal structures include the N-terminal domains of *S. solfataricus*, *M. thermoautotrophicum*, and *Thermoplasma acidophilum* MCM proteins, with the latter forming a right-handed spiral filament (Fletcher et al., 2003; Liu et al., 2008; Fu et al., 2014). A left-handed filament structure for near full-length *S. solfataricus* MCM has also been determined (Slaymaker et al., 2013), as well as a full-length structure of a catalytically inactive MCM homolog from *Methanopyrus kandleri* (Bae et al., 2009). The biological significance, if any, of the filamentous forms remains to be determined.

Unlike the MCM proteins, archaeal GINS and Cdc45 homologs share only very limited sequence similarity with their eukaryotic counterparts (Marinsek et al., 2006). The eukaryotic GINS complex is a heterotetramer, comprising the related Sld5, Psf1, Psf2, and Psf3 proteins (reviewed by Kamada, 2012). Both homotetrameric and heterotetrameric (i.e., dimer of dimer or A_2_B_2_) complexes have been identified in archaea and the structure of the *T. kodakarensis* A_2_B_2_ heterotetrameric GINS has been solved (Oyama et al., 2011). Archaeal Cdc45 homologs have only very recently been positively identified as such (Sanchez-Pulido and Ponting, 2011; Krastanova et al., 2012; Makarova et al., 2012). These proteins belong to the RecJ nuclease branch of the DHH hydrolase superfamily. Unlike eukaryotic Cdc45 proteins, at least some archaeal RecJ/Cdc45 proteins possess nuclease activity (Li et al., 2011; Yuan et al., 2013), the precise function of which is unclear. The existence of all three CMG components in archaea suggest that these organisms may have a valuable role to play as models for dissecting the function of the individual CMG components.

Using multiple sequence alignments and crystal structures as a guide, a number of laboratories have reported detailed mutagenesis studies of MCM structure-function relationships (reviewed by Slaymaker and Chen, 2012). In all cases, the effects of the mutations on MCM function were determined *in vitro*, using purified recombinant proteins in various biochemical assays. To our knowledge, there has been no *in vivo* reverse genetic analysis of the effects of mutations of MCM function, largely due to the difficulty or impossibility of conducting such studies in species where genetic tools are either rudimentary or unavailable. In this report we describe the first results of reverse genetic analysis of archaeal MCM function *in vivo*, using the haloarchaeal organism *Haloferax volcanii* as a model system. The haloarchaea present a particularly attractive model to study archaeal chromosome replication owing to the ease with which representative species can be manipulated genetically (reviewed by Farkas et al., 2013). *Hfx. volcanii* in particular has proved a highly successful model, with a number of components of the *Hfx. volcanii* replication machinery already characterized, including multiple origins of replication (Hawkins et al., 2013), origin binding proteins (Norais et al., 2007), single-stranded DNA binding proteins (Skowyra and MacNeill, 2012; Stroud et al., 2012), the sliding clamp PCNA (Morgunova et al., 2009; Winter et al., 2009) and both ATP- and NAD-dependent DNA ligases (Poidevin and MacNeill, 2006; Zhao et al., 2006). In addition to their genetic tractability, over 100 haloarchaeal genomes have been now sequenced, offering a wealth of information for comparative protein sequence analysis. *Hfx. volcanii* encodes a single MCM protein, a member of the previously defined core group of haloarchaeal MCM proteins discussed further below (MacNeill, 2009). In the work presented here, three conserved features of the protein are targeted for mutagenesis: the β7-β8 and β9-β10 β-hairpin loops and the four conserved cysteines of the putative zinc binding domain. The results of these studies establish *Hfx. volcanii* as a valuable model for detailed structure-function analysis of MCM helicase and provide confirmation of the importance of these conserved features for MCM function *in vivo*.

## Materials and methods

### Database searching and sequence handling

Protein sequences were obtained from the UniProt Knowledgebase (UniProtKB) database (Magrane and UniProt Consortium, 2011): primary accession numbers are listed in Table 1. Multiple sequence alignments and phylogenetic trees were generated using ClustalX 2.1 (Larkin et al., 2007) and njplot (Perriere and Gouy, 1996), respectively. Intein sequences were initially identified by visual inspection as large sequence insertions in comparative sequence analysis. Inteins boundaries were defined by the presence of the N-terminal (block A) and C-terminal (block G) intein splicing motifs as defined at the InBase intein database (Perler, 2002).

**Table 1:** **List of MCM proteins analyzed in this study**.

**Key to** Figure 1	**Species**	**UniProtKB accession number**	**Length (amino acids)**	**Inteins**			
				**A**	**B**	**C**	**D**
1	*Haladaptatus paucihalophilus* DX253	E7QNU9	698				
2	*Halalkalicoccus jeotgali* B3	D8J3U5	700				
3A	*Haloarcula marismortui* ATCC 43049	Q5UYX8	1175	+			
3B		Q5V011	681				
3C		Q5V814	649				
4	*Halobacterium salinarum* R1	B0R796	879	+			
5A	*Halobiforma lacisalsi* AJ5	M0LR18	1342			+	
5B		M0LZ47	312				
6	*Halococcus thailandensis* JCM 13552	M0N8X7	698				
7	*Haloferax volcanii* DS2	D4GZG5	702				
8	*Halogeometricum borinquense* DSM 11551	E4NRK9	1818	+		+	
9	*Halogranum salarium* B-1	J2ZGA0	700				
10A	*Halomicrobium mukohataei* DSM 12286	C7P1F9	873	+			
10B		C7NZ81	458				
11A	*Halopiger xanaduensis* SH-6	F8D3Z0	702				
11B		F8DEM3	698				
11C		F8DET6	315				
12	*Haloquadratum walsbyi* HBSQ001	Q18E84	2216	+	+	+	+
13	*Halorhabdus utahensis* AX-2	C7NUH7	1412	+		+	
14A	*Halorubrum lacusprofundi* DSM 5036	B9LTB1	700				
14B		B9LUI3	717				
15	*Halosarcina pallida* JCM 14848	M0D6S0	1172	+			
16A	*Halosimplex carlsbadense* 2-9-1	M0D2C8	698				
16B		M0CE93	712				
17A	*Haloterrigena turkmenica* DSM 5511	D2RUS4	700				
17B		D2S3H9	314				
18	*Halovivax asiaticus* JCM 14624	M0BKP0	876	+			
19A	*Natrialba asiatica* DSM 12278	M0AKM2	700				
19B		M0AIF1	315				
20	*Natrinema pallidum* DSM 3751	L9Z845	700				
21A	*Natronobacterium gregoryi* SP2	L0AHL1	1172	+			
21B		L0AN78	709				
21C		L0ANL5	662				
22	*Natronococcus amylolyticus* DSM 10524	L9XCC3	1404	+	+		
23	*Natronolimnobius innermongolicus* JCM 12255	L9X2E8	700				
24A	*Natronomonas pharaonis* DSM 2160	Q3IML4	1037	+			
24B		Q3IPB6	676				
25	*Natronorubrum tibetense* GA33	L9VHE4	1814	+		+	
26	*Salinarchaeum* sp. Harcht-Bsk1	R4W989	697				
	*Methanosarcina acetivorans* C2A	Q8TSW4	701				
	*Methanothermobacter thermautotrophicus* ΔH	O27798	666				
	*Sulfolobus solfataricus* P2	Q9UXG1	686				
	*Cenarchaeum symbiosum* A	A0RYB8	697				
	*Korarchaeum cryptofilum* OPF8	B1L6L9	703				
	*Thermoplasma acidophilum* DSM 1728	Q9HK10	698				
	*Archaeoglobus fulgidus* DSM 4304	O29733	586				
	*Homo sapiens* Mcm2	P49736	904				
	*Homo sapiens* Mcm3	P25205	808				
	*Homo sapiens* Mcm4	P33991	863				
	*Homo sapiens* Mcm5	P33992	734				
	*Homo sapiens* Mcm6	Q14566	821				
	*Homo sapiens* Mcm7	P33993	719				

**Figure 1:**
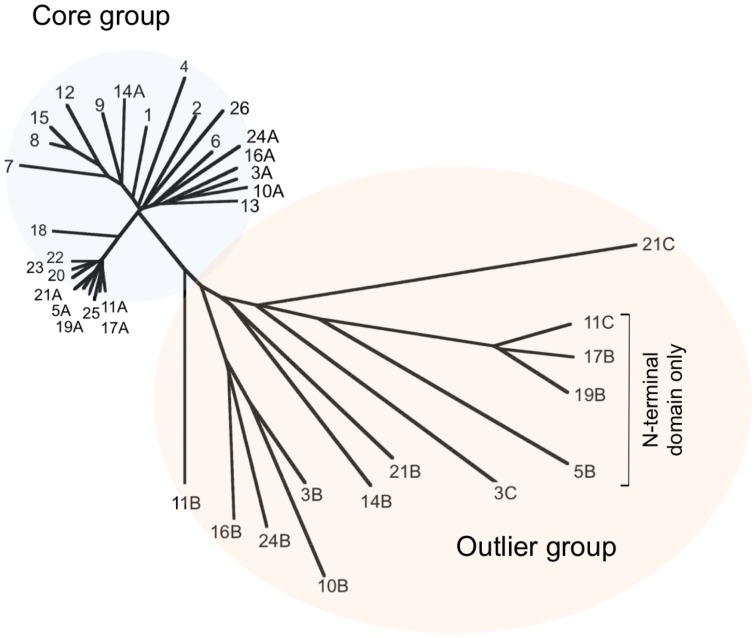
**Phylogenetic tree of representative haloarchaeal MCM proteins**. The sequences of 39 proteins from 26 haloarchaeal genera were compared using ClustalX 2.1 (Larkin et al., 2007) and an unrooted phylogenetic tree generated using njplot (Perriere and Gouy, 1996). Twenty-six proteins (one from each included species) form a core group with the remaining 13 proteins designated as outliers. Individual proteins are numbered according to Table 1. Accession numbers can also be found in Table 1.

**Figure 2:**
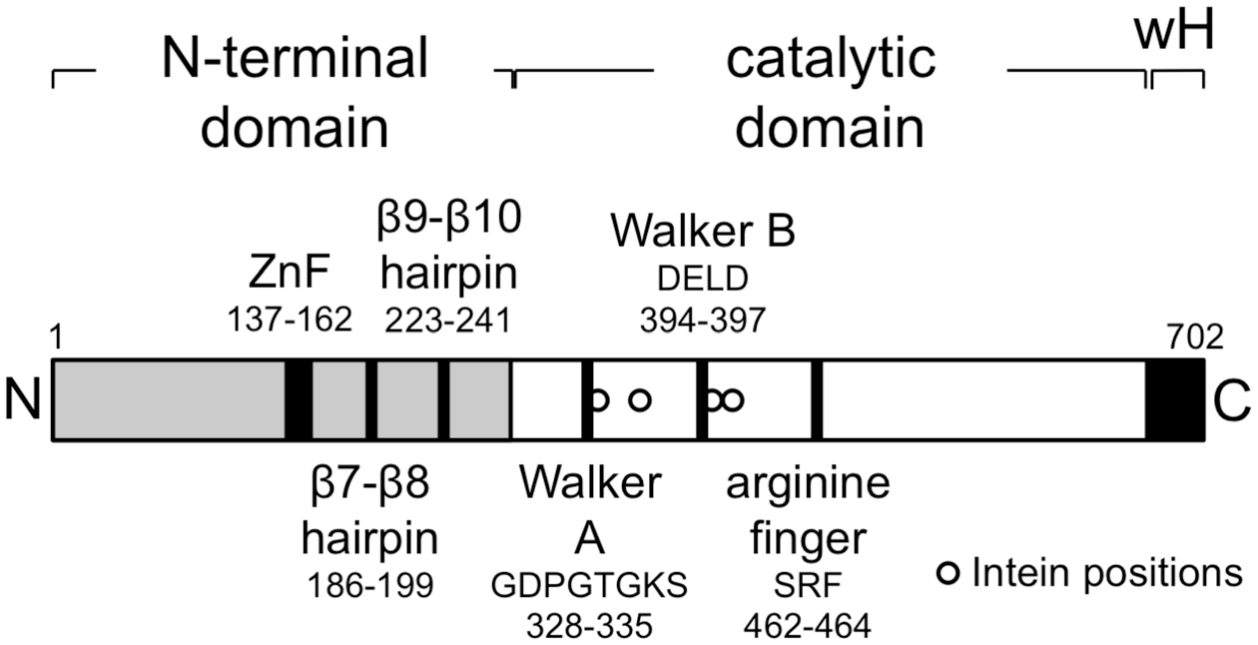
**The *Haloferax volcanii* MCM protein**. Schematic representation showing domain organization of the *Hfx. volcanii* MCM protein, highlighting the location of key structural motifs. The locations of intein insertions (from right to left, inteins A–D, see Table 1) in other haloarchaeal MCM proteins (not in *Hfx. volcanii* MCM, which is intein-free) are indicated by the open circles at positions 335, 362, 401, and 415.

*The numbers shown in the first column provide a key for the proteins represented in the phylogenetic tree shown in Figure 1. The plus signs in the final four columns indicate the present of intein insertions at each of the four conserved positions A–D shown in Figure 2. Protein 3A, for example, Q5UYX8 from Haloarcula marismortui, has a single intein at position A*.

### Strains and growth conditions

*Hfx. volcanii* strains used in this study are listed in Table 2. All strains were grown in either Hv-YPC or Hv-CA medium at 45°C as described in the Halohandbook v7.2 (www.haloarchaea.com/resources/halohandbook). For selection procedures, tryptophan was added to Hv-CA medium at a final concentration of 50 μg/ml. For counter-selection using 5-fluoroorotic acid (5-FOA), Hv-CA was supplemented with uracil and 5-FOA at final concentrations of 10 μg/ml and 50 μg/ml, respectively. For mitomycin C sensitivity assays, wild-type (H26) and mutant cells were grown at 45°C in Hv-YPC medium to an OD_650 nm_ of 0.2–0.32, before being serially diluted in 18% SW. 5 μl aliquots were then spotted on Hv-YPC plates containing 0, 10, 20, or 30 ng/ml mitomycin C and incubated at 45°C for 5 days.

**Table 2:** ***Haloferax volcanii* strains used in this study**.

**Strain no**.	**Genotype**	**Notes**	**References**
SMH693	–	Wild-type strain DS70	Wendoloski et al., 2001
SMH630	*ΔpyrE2*	Strain H26	Allers et al., 2004
SMH656	*mcm-rM1 ΔpyrE2*	Single alanine substitution Q186A	This study
SMH654	*mcm-rM3 ΔpyrE2*	Single alanine substitution E190A	This study
SMH658	*mcm-rM4 ΔpyrE2*	Single alanine substitution R193A	This study
SMH660	*mcm-rM5 ΔpyrE2*	Single alanine substitution E196A	This study
SMH662	*mcm-rM6 ΔpyrE2*	Single alanine substitution Q199A	This study
SMH638	*mcm-bH1 ΔpyrE2*	Paired alanine substitution H226A/I227A	This study
SMH640	*mcm-bH2 ΔpyrE2*	Paired alanine substitution E228A/Q229A	This study
SMH642	*mcm-bH3 ΔpyrE2*	Paired alanine substitution Q230A/T231A	This study
SMH644	*mcm-bH4 ΔpyrE2*	Paired alanine substitution S232A/G233A	This study
SMH646	*mcm-bH5 ΔpyrE2*	Paired alanine substitution N234A/E235A	This study
SMH648	*mcm-bH6 ΔpyrE2*	Paired alanine substitution K236A/T237A	This study
SMH649	*mcm-hD1 ΔpyrE2*	Two amino acid deletion T231/S232	This study
SMH650	*mcm-hD2 ΔpyrE2*	Four amino acid deletion Q230/T231/S232/G233	This study
SMH652	*mcm-S1 ΔpyrE2*	Silent *Acc*65I restriction site replacing codons 141 and 142	This study

### Molecular cloning reagents

Enzymes for molecular cloning were purchased from New England Biolabs (NEB), Promega or Fermentas. Oligonucleotides were synthesized by DNA Technology A/S (Risskov, Denmark). DNA sequencing was performed by Eurofins MWG Operon (Ebersberg, Germany). DNA purification kits were from Qiagen. PCR amplification was performed using the GC-rich PCR system (Roche) with *Taq* polymerase (NEB) substituting for the GC-rich enzyme as necessary. For routine cloning purposes, *E.coli* DH5α (*fhuA2* Δ*(argF-lacZ)U169 phoA glnV44 Φ 80* Δ*(lacZ)M15 gyrA96 recA1 relA1 endA1 thi-1 hsdR17*) was used (Invitrogen). To prepare unmethylated plasmid DNA for *Hfx. volcanii* transformation, *E.coli* GM121 (F- *dam-3 dcm-6 ara-14 fhuA31 galK2 galT22 hdsR3 lacY1 leu-6 thi-1 thr-1 tsx-78*) was used.

### Construction of mutant *Hfx. volcanii* strains

Mutant strains were constructed using the pop-in/pop-out method (Bitan-Banin et al., 2003) in *Hfx. volcanii ΔpyrE2* strain H26 as follows. First, plasmid pTA131-HfxMCM-HXba was constructed by using oligonucleotide primers HfxMCM-5H and HfxMCM-3X (designed to include *Hin*dIII and *Xba*I sites respectively, see Table 3) to amplify a 1.0 kb fragment of *Hfx. volcanii* genomic DNA spanning the region from 100 nucleotides upstream of the *mcm* ORF to 900 nucleotides inside the ORF. The PCR product was digested with *Hin*dIII and *Xba*I, cloned into plasmid pTA131 digested with the same two enzymes (Allers et al., 2004) and sequenced to confirm the absence of PCR errors.

**Table 3:** **Oligonucleotides used in this study**.

**Primer name**	**Sequence**
**A. FOR CONSTRUCTION OF pTA131-HfxMCM-HXba**	
HfxMCM-5H	5'-GTGTGTGTGTAAGCTTCCTCCGCGAGGCGACGGA-3'
HfxMCM-3X	5'-GGTGGTGGTGTCTAGACATGGCAATCTTCTCCTG-3'
**B. FOR CONSTRUCTION OF β7-β8 β-HAIRPIN MUTANTS BY OEM**	
HFXMCM-rM1-F HFXMCM-rM1-R	5'-AAACTGCGCGTCGCCGAGTCCCCCGAGGGC-3' 5'-GCCCTCGGGGGACTCGGCGACGCGCAGTTT-3'
HFXMCM-rM2-F HFXMCM-rM2-R	5'-AAACTGCGCGTCCAGGCCTCCCCCGAGGGCCTGCG-3' 5'-CGCAGGCCCTCGGGGGAGGCCTGGACGCGCAGTTT-3'
HFXMCM-rM3-F HFXMCM-rM3-R	5'-CAGGAGTCCCCCGCCGGCCTGCGCGGGGGC-3' 5'-GCCCCCGCGCAGGCCGGCGGGGGACTCCTG-3'
HFXMCM-rM4-F HFXMCM-rM4-R	5'-CCCGAGGGCCTGGCGGGGGGCGAGACGCCG-3' 5'-CGGCGTCTCGCCCCCCGCCAGGCCCTCGGG-3'
HFXMCM-rM5-F HFXMCM-rM5-R	5'-CTGCGCGGGGGCGCCACGCCGCAGAGCATC-3' 5'-GATGCTCTGCGGCGTGGCGCCCCCGCGCAG-3'
HFXMCM-rM6-F HFXMCM-rM6-R	5'-GGCGAGACGCCGGCCAGCATCGACATCAAC-3' 5'-GTTGATGTCGATGCTGGCCGGCGTCTCGCC-3'
HFXMCM-rD1-F HFXMCM-rD1-R	5'-CAGGAGTCCCCCGAG-GGCGAGACGCCGCAG-3' 5'-CTGCGGCGTCTCGCC-CTCGGGGGACTCCTG-3'
HFXMCM-rD2-F HFXMCM-rD2-R	5'-GTCCAGGAGTCCCCC-GAGACGCCGCAGAGC-3' 5'-GCTCTGCGGCGTCTC-GGGGGACTCCTGGAC-3'
HFXMCM-rD3-F HFXMCM-rD3-R	5'-CGCGTCCAGGAGTCC-ACGCCGCAGAGCATC-3' 5'-GATGCTCTGCGGCGT-GGACTCCTGGACGCG-3'
HFXMCM-rD4-F HFXMCM-rD4-R	5'-CTGCGCGTCCAGGAG-CCGCAGAGCATCGAC-3' 5'-GTCGATGCTCTGCGG-CTCCTGGACGCGCAG-3'
**C. FOR CONSTRUCTION OF β9-β10 β-HAIRPIN MUTANTS BY OEM**	
HFXMCM-bH1-F HFXMCM-bH1-R	5'-GTCGGCGTCCTCGCAGCGGAACAGCAGACATCG-3' 5'-CGATGTCTGCTGTTCCGCTGCGAGGACGCCGAC-3'
HFXMCM-bH2-F HFXMCM-bH2-R	5'-GTCCTCCACATCGCAGCGCAGACATCGGGCAAC-3' 5'-GTTGCCCGATGTCTGCGCTGCGATGTGGAGGAC-3'
HFXMCM-bH3-F HFXMCM-bH3-R	5'-CACATCGAACAGGCAGCGTCGGGCAACGAGAAG-3' 5'-CTTCTCGTTGCCCGACGCTGCCTGTTCGATGTG-3'
HFXMCM-bH4-F HFXMCM-bH4-R	5'-GAACAGCAGACAGCAGCGAACGAGAAGACGCCC-3' 5'-GGGCGTCTTCTCGTTCGCTGCTGTCTGCTGTTC-3'
HFXMCM-bH5-F HFXMCM-bH5-R	5'-CAGACATCGGGCGCAGCGAAGACGCCCGTCTTC-3' 5'-GAAGACGGGCGTCTTCGCTGCGCCCGATGTCTG-3'
HFXMCM-bH6-F HFXMCM-bH6-R	5'-TCGGGCAACGAGGCAGCGCCCGTCTTCGACTAC-3' 5'-GTAGTCGAAGACGGGCGCTGCCTCGTTGCCCGA-3'
HFXMCM-hD1-F HFXMCM-hD1-R	5'-CACATCGAACAGCAG-GGCAACGAGAAGACG-3' 5'-CGTCTTCTCGTTGCC-CTGCTGTTCGATGTG-3'
HFXMCM-hD2-F HFXMCM-hD2-R	5'-CTCCACATCGAACAG-AACGAGAAGACGCCC-3' 5'-GGGCGTCTTCTCGTT-CTGTTCGATGTGGAG-3'
HFXMCM-hD3-F HFXMCM-hD3-R	5'-GTCCTCCACATCGAA-GAGAAGACGCCCGTC-3' 5'-GACGGGCGTCTTCTC-TTCGATGTGGAGGAC-3'
HFXMCM-hD4-F HFXMCM-hD4-R	5'-GGCGTCCTCCACATC-AAGACGCCCGTCTTC-3' 5'-GAAGACGGGCGTCTT-GATGTGGAGGACGCC-3'
**D. FOR CONSTRUCTION OF ZINC BINDING DOMAIN MUTANTS BY OEM**	
HFXMCM-C137A-F HFXMCM-C137A-R	5'-CCGCCTTCGAGGCGCAGCGCTGCGG-3' 5'-CCGCAGCGCTGCGCCTCGAAGGCGG-3'
HFXMCM-C140A-F HFXMCM-C140A-R	5'-AGTGCCAGCGCGCGGGGACGATGAG-3' 5'-CTCATCGTCCCCGCGCGCTGGCACT-3'
HFXMCM-C159A-F HFXMCM-C159A-R	5'-AACCCCACGAGGCGCAGGGATGCGA-3'5'-TCGCATCCCTGCGCCTCGTGGGGTT-3'
HFXMCM-C162A-F HFXMCM-C162A-R	5'-AGTGTCAGGGAGCGGAGCGCCAGGG-3' 5'-CCCTGGCGCTCCGCTCCCTGACACT-3'
**E. FOR CONSTRUCTION OF SILENT Acc65I mutation by OEM**	
HFXMCM-S1-F HFXMCM-S1-R	5'-TGCCAGCGCTGCGGTACCATGAGCTACATC-3' 5'-GATGTAGCTCATGGTACCGCAGCGCTGGCA-3'
**F. FOR DETECTION OF β7-β8 β-HAIRPIN MUTANTS**	
HFXMCM-rM1-WT	5'-CAGAAACTGCGCGTCCAG-3'
HFXMCM-rM1-MUT	5'-CAGAAACTGCGCGTCGCC-3'
HFXMCM-rM2-WT	5'-AAACTGCGCGTCCAGGAG-3'
HFXMCM-rM2-MUT	5'-AAACTGCGCGTCCAGGCC-3'
HFXMCM-rM3-WT	5'-GTCCAGGAGTCCCCCGAG-3'
HFXMCM-rM3-MUT	5'-GTCCAGGAGTCCCCCGCC-3'
HFXMCM-rM4-WT	5'-TCCCCCGAGGGCCTGCGC-3'
HFXMCM-rM4-MUT	5'-TCCCCCGAGGGCCTGGCG-3'
HFXMCM-rM5-WT	5'-GGCCTGCGCGGGGGCGAG-3'
HFXMCM-rM5-MUT	5'-GGCCTGCGCGGGGGCGCC-3'
HFXMCM-rM6-WT	5'-GGGGGCGAGACGCCGCAG-3'
HFXMCM-rM6-MUT	5'-GGGGGCGAGACGCCGGCC-3'
HFXMCM-rD1-MUT	5'-GGAGTCCCCCGAGGGCGAG-3'
HFXMCM-rD2-MUT	5'-CCAGGAGTCCCCCGAGAC-3'
HFXMCM-rD3-MUT	5'-CGTCCAGGAGTCCACGCC-3'
HFXMCM-rD4-MUT	5'-CTGCGCGTCCAGGAGCCG-3'
**G. FOR DETECTION OF β9-β10 β-HAIRPIN MUTANTS**	
HFXMCM-bH1-WT	5'-GTCGGCGTCCTCCACATC-3'
HFXMCM-bH1-MUT	5'-GTCGGCGTCCTCGCAGCG-3'
HFXMCM-bH2-WT	5'-GTCCTCCACATCGAACAG-3'
HFXMCM-bH2-MUT	5'-GTCCTCCACATCGCAGCG-3'
HFXMCM-bH3-WT	5'-CACATCGAACAGCAGACA-3'
HFXMCM-bH3-MUT	5'-CACATCGAACAGGCAGCG-3'
HFXMCM-bH4-WT	5'-GAACAGCAGACATCGGGC-3'
HFXMCM-bH4-MUT	5'-GAACAGCAGACAGCAGCG-3'
HFXMCM-bH5-WT	5'-CAGACATCGGGCAACGAG-3'
HFXMCM-bH5-MUT	5'-CAGACATCGGGCGCAGCG-3'
HFXMCM-bH6-WT	5'-ACATCGGGCAACGAGAAGA-3'
HFXMCM-bH6-MUT	5'-ACATCGGGCAACGAGGCAG-3'
HFXMCM-hD1-WT	5'-CACATCGAACAGCAGACA-3'
HFXMCM-hD1-MUT	5'-CACATCGAACAGCAGGGC-3'
HFXMCM-hD2-WT	5'-CTCCACATCGAACAGCAG-3'
HFXMCM-hD2-MUT	5'-CTCCACATCGAACAGAAC-3'
HFXMCM-hD3-WT	5'-TCCTCCACATCGAACAGC-3'
HFXMCM-hD3-MUT	5'-TCCTCCACATCGAAGAGA-3'
HFXMCM-hD4-WT	5'-GGCGTCCTCCACATCGAA-3'
HFXMCM-hD4-MUT	5'-GGCGTCCTCCACATCAAG-3'
**H. FOR DETECTION OF ZINC BINDING DOMAIN MUTANTS**	
HFXMCM-C137A-WT	5'-GAAGCCGCCTTCGAGTGC-3'
HFXMCM-C137A-MUT	5'-GAAGCCGCCTTCGAGGCG-3'
HFXMCM-C140A-WT	5'-TTCGAGTGCCAGCGCTGC-3'
HFXMCM-C140A-MUT	5'-TTCGAGTGCCAGCGCGCG-3'
HFXMCM-C159A-WT	5'-CAGGAACCCCACGAGTGT-3'
HFXMCM-C159A-MUT	5'-CAGGAACCCCACGAGGCG-3'
HFXMCM-C162A-WT	5'-CACGAGTGTCAGGGATGC-3'
HFXMCM-C162A-MUT	5'-CACGAGTGTCAGGGAGCG-3'
**I. REVERSE PRIMER FOR MUTANT DETECTION**	
HFXMCM-R1150	5'-GCGAATCCGCGAGCCGTC-3'

*Restriction sites in oligonucleotide primers used for pTA131-HfxMCM-HXba construction underlined. Oligonucleotide primers for overlap extension mutagenesis (OEM) are shown in top strand-bottom strand pairs with the mutated bases underlined in the top strand primer (for amino acid substitutions) or with deletion boundaries indicated with a dash. Oligonucleotide primers for mutant detection are shown with 3 ' mismatched bases underlined*.

The resulting plasmid (pTA131-HfxMCM-HXba) was then used as a template for PCR overlap extension mutagenesis (OEM) to create 25 mutant derivatives. The sequences of the mutagenic primers are given in Table 3. OEM was performed using the GC-Rich PCR system buffer and GC-Rich enzyme mix (Roche) for the first round of PCR and the GC-Rich PCR system buffer and *Taq* polymerase (NEB) for the second round. Oligonucleotides HfxMCM-5H and HfxMCM-3X were used as the flanking primers throughout. The resulting PCR products were then restricted with *Bsu*36I and *Bst*EII (for β7-β8 β-hairpin and zinc binding domain mutants) or *Bst*EII and *Xba*I (for β9-β10 β-hairpin mutants) and the 329 bp *Bsu*36I-*Bst*EII or 271 bp *Bst*EII-*Xba*I pieces carrying the mutations cloned back into pTA131-HfxMCM-HXba from which the corresponding wild-type restriction fragments had been removed. Plasmids were again sequenced to confirm the absence of unwanted sequence changes before being passaged through *E.coli* GM121 to generate unmethylated DNA for transformation into *Hfx. volcanii ΔpyrE2* strain H26 (Table 2).

Transformation of *Hfx.volcanii* was accomplished as described in the Halohandbook v7.2 (www.haloarchaea.com/resources/halohandbook). Transformants obtained on Hv-CA medium lacking uracil were grown for 30 generations at 45°C in non-selective Hv-YPC medium to allow loss of the plasmid before being plated on Hv-CA plates containing 50 μg/ml 5-FOA and a 10 μg/ml uracil. Colonies formed on these plates were then inoculated into 500 μl of Hv-YPC liquid medium and grown overnight at 45°C. Genomic DNA from these was prepared by taking 10 μl of overnight culture, adding to 500 μl of sterile water and heating to 70°C for 10 min to lyse the cells. For the β-hairpin and zinc binding domain mutants, 1 μl of the resulting mix was used in PCR reactions to screen for the presence of the mutants in the chromosome. To discriminate between wild-type and mutant sequences, oligonucleotide primers with mismatched 3' sequences were used in PCR reactions in conjunction with primer HFXMCM-R1150, which lies 1150 nucleotides into the *mcm* ORF and which is therefore not present in pTA131-HfxMCM-HXba (see Table 3 for oligonucleotide sequences). For the silent restriction site mutant, the partial *mcm* ORF was amplified using primers HfxMCM-5H and HfxMCM-3X and the PCR products digested with *Acc*65I to identify mutants. In all cases, putative positive clones were then re-streaked twice to single colonies on Hv-YPC agar at 45°C before being re-tested by PCR using wild-type and mutant primers. Finally, the presence of the mutation was confirmed by sequencing of the relevant part of the *mcm* ORF amplified by PCR from genomic DNA (see Supplementary Information for DNA sequence traces).

## Results

### Haloarchaeal MCM proteins

Prior to embarking on reverse genetic analysis of *Hfx. volcanii* MCM protein function, we undertook a detailed bioinformatic analysis of MCM protein distribution and conservation across the haloarchaea. To identify haloarchaeal MCM proteins, we searched the UniProtKB sequence database using BLAST with the *Hfx. volcanii* MCM protein (HVO_0220, UniProtKB accession number D4GZG5) as the query sequence. Almost 200 proteins were identified in ~120 species belonging to 26 different genera of the *Halobacteriaceae*. To simplify further analysis, we selected a single species as a representative of each genus. These 26 species encoded a total of 39 MCM proteins. A complete list of the species under investigation, together with accession numbers for the proteins we identified, can be found in Table 1.

To investigate the relationship between the proteins in greater detail, we constructed multiple protein sequence alignments using ClustalX 2.1 (Larkin et al., 2007). Previously, when analysing a significantly smaller number of genomes (five), we identified a core group of five MCM proteins and a further three proteins that we designated as outliers (MacNeill, 2009). We performed a similar analysis of the larger dataset, using ClustalX to generate multiple sequence alignments of protein sequences (from which inteins were first removed—see below) and njplot (Perriere and Gouy, 1996) to generate unrooted phylogenetic trees. Figure 1 shows the phylogenetic tree for the 39 proteins. As before, two groups are apparent, corresponding to the core and outlier groups defined previously (MacNeill, 2009). Each of the 26 species encodes a single member of the core group that includes the *Hfx. volcanii* MCM protein. The core group MCM proteins range in length from 697 to 702 amino acids (after inteins are removed—see below) and display a minimum pairwise protein sequence identity of 68% (range 68–95%). Three hundred and twenty four residues (46% of the protein sequence) are absolutely conserved across all 26 core group proteins. This includes the key catalytic residues that make up the Walker A (P-loop) motif (GDPGTGKS in all 26 proteins compared to the consensus GX_4_GKS/T), the Walker B motif (DELD in all 26 proteins) and arginine finger motif (SRF in all 26 proteins).

The outlier group proteins display much greater variety in length (312–717 amino acids) and sequence similarity (24–48% identity with *Hfx. volcanii* MCM). The four shortest proteins (312–315 amino acids) are made up of sequences related to the non-catalytic N-terminal domain of MCM only and cannot possess helicase activity. The remaining nine outliers all possess conserved Walker B (DEL/ID) and arginine finger (SRF) motifs, as well as the key lysine in the Walker A (P-loop) motif, suggesting that these proteins may well have ATPase and/or helicase activities. To date, none has been characterized biochemically.

Inteins are parasitic genetic elements capable of efficient self-splicing at the protein level (Gogarten and Hilario, 2006) and are a common feature of the haloarchaeal MCM proteins. Amongst the 39 proteins, inteins were found in 13, all members of the core group defined above, and at four different locations within the C-terminal catalytic domain of the protein, with the largest number of inteins in an individual MCM protein being four (Table 1). Figure 2 shows the position of the four inteins relative to conserved sequence features found in archaeal MCM proteins. As noted previously, inteins are frequently located at or very near to highly conserved and functionally important sequence regions (Gogarten and Hilario, 2006) and the haloarchaeal MCM inteins are no exception: intein insertion site A is located immediately C-terminal to the essential lysine in the Walker A sequence GDPGTGKS mentioned above, while intein C lies just four amino acids C-terminal to the Walker B sequence DELD (Table 1, Figure 2). Intein splicing is therefore likely to be essential for protein function.

In a number of archaeal organisms, including representatives of the crenarchaea, euryarchaea, thaumarchaea and korarchaea, the gene encoding MCM is found adjacent to that encoding a GINS subunit (MacNeill, 2010). To ask whether this arrangement is also found in any of the species under investigation in this report, we examined the chromosomal context of each of the 26 core MCM genes and also the 13 outlier proteins. None of the genes encoding core group proteins is located adjacent to a gene encoding GINS or indeed, to any known replication gene (data not shown). A similar situation is seen with genes encoding 12 of the 13 outlier proteins, the sole exception being the pNG3053 protein (UniProtKB accession number Q5V814, labeled 3C in Figure 1) encoded by plasmid pNG300 of *Haloarcula marismortui* which is located immediately 3' to an ORF encoding the C-terminally truncated GINS homolog pNG3052 (data not shown).

### Reverse genetic analysis of MCM function

*Hfx. volcanii* encodes a single intein-free MCM protein of 702 amino acids in length (Table 1) that comprises an N-terminal domain that spans residues 1–283, an AAA+ catalytic core domain spanning residues 283–632 and a C-terminal winged helix-turn-helix (wHTH) domain spanning residues 633–702. All the conserved sequence features characteristic of archaeal MCM proteins are found in the *Hfx. volcanii* protein (Figure 2). In order to test whether *Hfx. volcanii* was a workable model for reverse genetic analysis of archaeal MCM function, three of these conserved features were targeted for mutagenesis: the β7-β8 β-hairpin loop (also known as the allosteric communication loop, ACL), the β9-β10 β-hairpin loop and the putative zinc binding domain (Figure 2).

To introduce mutations into the *mcm* gene, we used the pop-in/pop-out method (Bitan-Banin et al., 2003). To achieve this, a plasmid was constructed carrying a 1.0 kb region of the *Hfx. volcanii* genome spanning the first 900 nucleotides of the *mcm* open reading frame together with 100 nucleotides of 5' flanking region. The plasmid also carries the *pyrE2* selectable marker (*pyrE2* function is required for uracil prototrophy) but does not possess a replication origin capable of promoting autonomous replication in *Hfx. volcanii*. Stable maintenance therefore requires that the plasmid integrates (pops-in) into the *Hfx. volcanii* genome by means of homologous recombination between the *mcm* sequences on the plasmid and the *mcm* gene in the chromosome. PCR overlap extension mutagenesis was used to generate a series of mutated forms of the plasmid in which the targeted amino acids were either replaced, singly or in pairs, with one or two alanine residues respectively, or deleted altogether. Next, the plasmids were introduced into *Hfx. volcanii ΔpyrE2* strain H26 by standard methods and transformant (pop-in) colonies obtained on Hv-CA plates lacking uracil. Multiple independent colonies were then individually picked and grown in non-selective (uracil-containing) Hv-YPC medium before being plated onto Hv-CA plates containing 5-fluoroorotic acid to select for (pop-out) clones that had lost the plasmid (see Materials and Methods). Pop-out colonies were then screened by PCR using oligonucleotide primers specific for either the wild-type or mutant sequences (see Table 3 for primer sequences). Candidate mutant strains were sequentially re-streaked twice on non-selective medium (Hv-YPC) before the presence of the mutation was confirmed by PCR using wild-type- and mutant-specific primers and by sequencing of amplified genomic DNA (see Materials and Methods). Table 2 lists the strains constructed by this method.

### Mutagenesis of the β7-β8 β-hairpin loop

**Figure 3:**
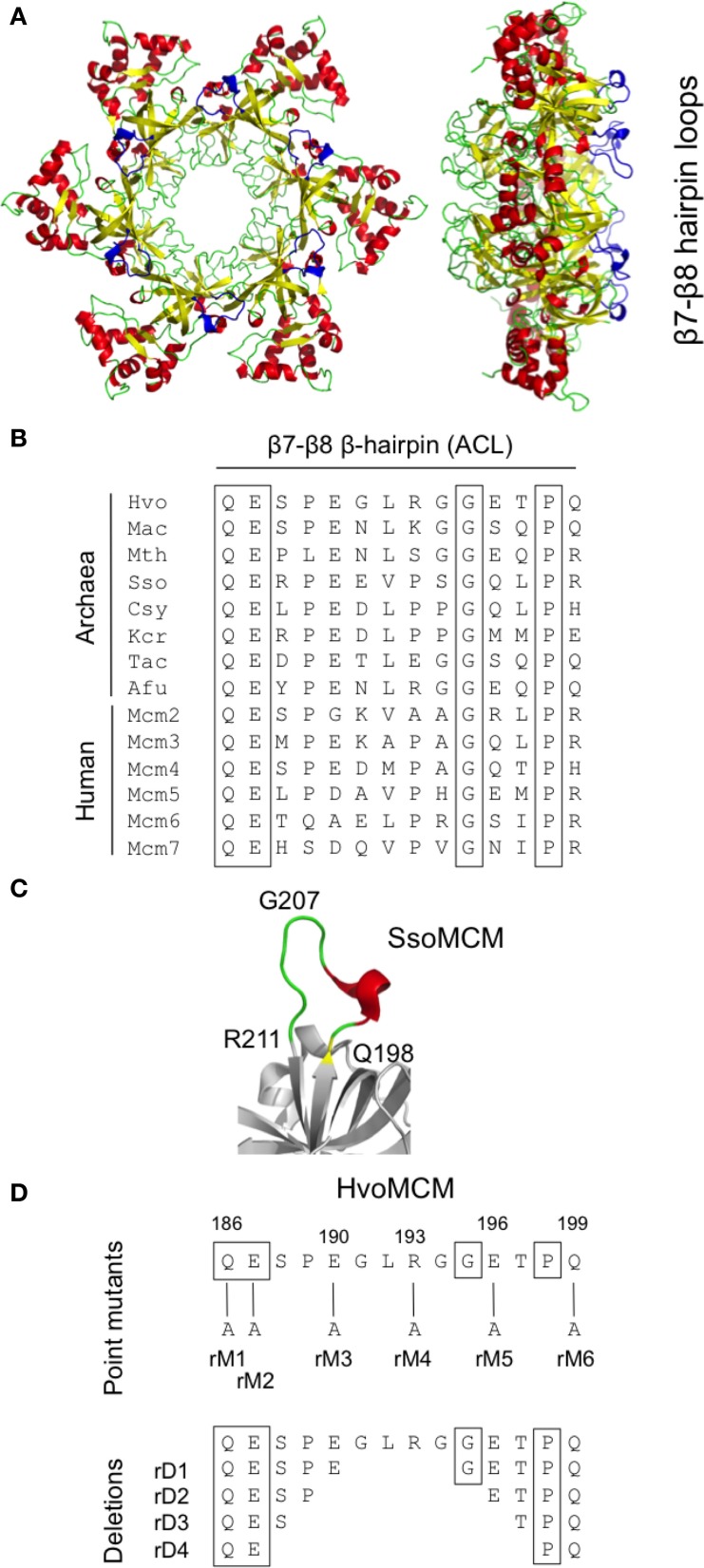
**Mutagenesis of the β7-β8 β-hairpin loop**. **(A)** Two views of the three-dimensional structure of the *S. solfataricus* MCM N-terminal domain hexamer (PDB entry 2VL6) with the β7-β8 β-hairpin loop colored in blue. **(B)** Alignment of β7-β8 β-hairpin loop region from MCM proteins from diverse archaeal species (Hvo, *Haloferax volcanii*; Mac, *Methanosarcina acetivorans*; Mth, *Methanothermobacter thermoautotrophicus*; Sso, *Sulfolobus solfataricus*; Csy, *Cenarchaeum symbiosum*; Kcr, *Korarchaeum cryptophilum*; Tac, *Thermoplasma acidophilum*; Afu, *Archaeoglobus fulgidus*) and from human. Conserved amino acids are boxed. Detailed strain designations and protein accession numbers can be found in Table 1. (**C)** Close-up view of β7-β8 β-hairpin loop in *S. solfataricus* MCM (PDB 2VL6). **(D)** Location and nature of intended mutations in *Hfx. volcanii* MCM protein. Attempts were made to construct six single amino acid substitutions (rM1–6) and four deletions (rD1–4). See text for details.

First identified on the basis of its high degree of sequence conservation across species, the β7-β8 β-hairpin loop (also known as the allosteric communication loop, ACL) is located at the interface between the N-terminal and AAA+ catalytic domains of the MCM protein (Figure 3). A number of mutants in β7-β8 loop have previously been analyzed biochemically, leading to the conclusion that the loop plays a role in coupling the activities of these two domains (Sakakibara et al., 2008; Barry et al., 2009). The mutations tested include six single point mutants in recombinant *M. thermoautotrophicum* MCM (including four at the boxed conserved residues shown in Figure 3B) as well as a triple point mutant and a complete replacement of the loop by the tripeptide serine-asparagine-glycine in *S. solfataricus* MCM (Sakakibara et al., 2008; Barry et al., 2009).

In order to test whether the function of the β7-β8 loop function was essential *in vivo* in *Hfx. volcanii*, we attempted to construct 10 different alleles (Figure 3): six single point mutants in which individual charged or bulky polar amino acids within the loop were replaced with alanine (mutants *mcm-rM1—mcm-rM6*) and four deletions, of two, four, six, and eight amino acids (mutants *mcm-rD1—mcm-rD4*, respectively). Two of the individual amino acids targeted (Q186 and E187) are conserved across species (Figure 3B); the equivalent residues were mutated in *M. thermoautotrophicum* MCM (Sakakibara et al., 2008).

**Figure 4:**
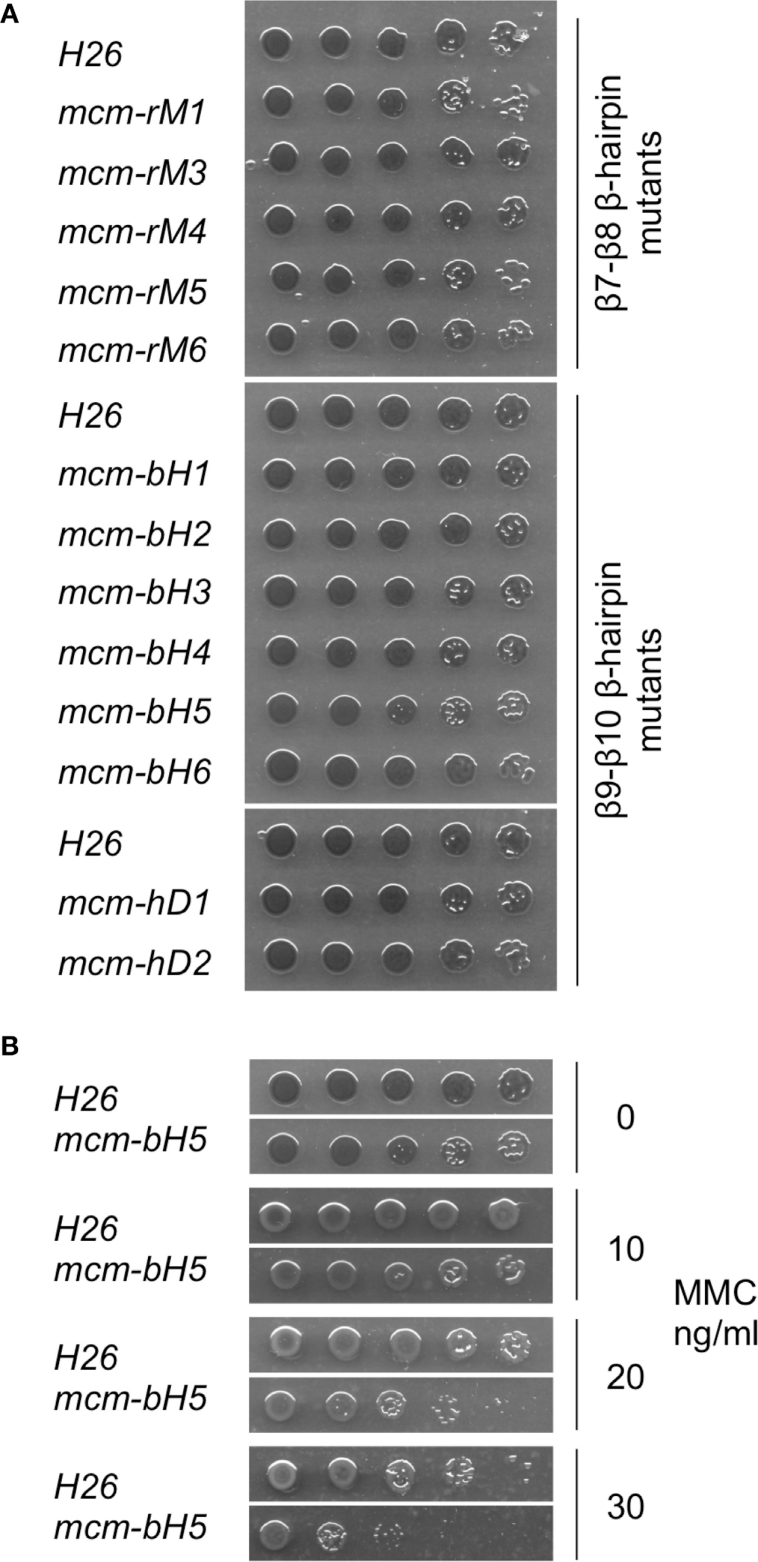
**Growth of mutant *mcm* strains**. Mutant and wild-type (H26) strains were grown to mid-log phase in Hv-YPC medium (OD_650 nm_ of 0.2–0.32) before being serially diluted in 18% SW and spotted onto Hv-YPC plates (part **A**) or Hv-YPC plates containing 0, 10, 20, or 30 ng/ml mitomycin C (MMC, part **B**, only H26 and *mcm-bH5* are shown). The plates were then incubated for 5 days at 45°C. β9-β10 β-hairpin loop mutant *mcm-bH5* is significantly more sensitive to MMC than wild-type (H26).

Of the 10 mutants, five (*mcm-rM1*, *mcm-rM3*—*mcm-rM6*) were readily identified by PCR screening using genomic DNA templates and oligonucleotide primers specific for the wild-type and mutant sequences (see Table 3) and confirmed by DNA sequencing (Supplementary Figure S1). Despite extensive screening (see Materials and methods), the remaining four strains (*mcm-rM2*, *mcm-rD1*—*mcm-rD4*) could not be isolated, suggesting that these mutations either inactivate or significantly impair the function of the *Hfx. volcanii* MCM protein in cells grown at 45°C. Growth of the five viable mutants was indistinguishable from the parental wild-type strain H26 (Figure 4A, upper panel). These results indicate that the β7-β8 loop is indeed essential for *Hfx. volcanii* MCM function although it is also clear that some point mutations (including replacement of conserved amino acid glutamine 186 with alanine) can be tolerated.

### Mutagenesis of the β9-β10 β-hairpin loop

**Figure 5:**
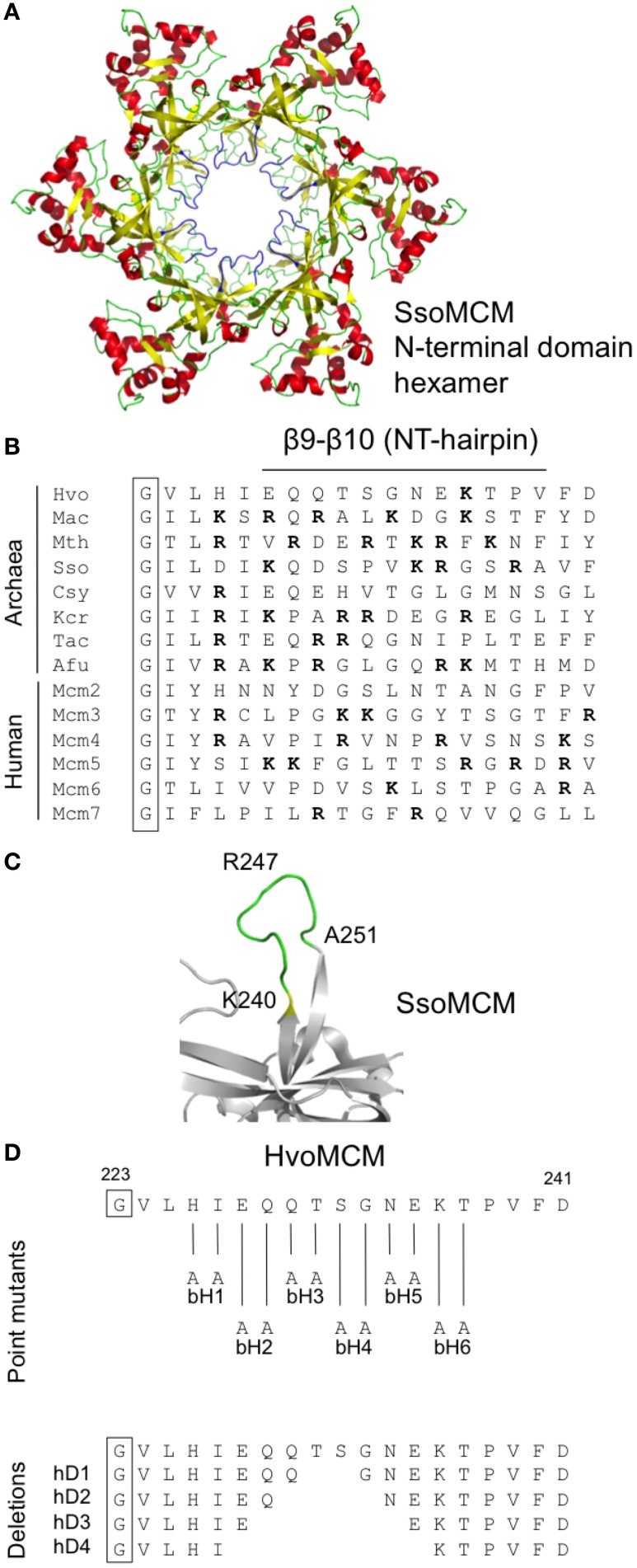
**Mutagenesis of the β9-β10 β-hairpin (NT-hairpin). (A)** Structure of the *S. solfataricus* MCM N-terminal domain hexamer (PDB 2VL6) with the β9-β10 β-hairpins highlighted in blue. **(B)** Multiple sequence alignment of the β9-β10 β-hairpin region from diverse archaeal species and from human (see legend to Figure 3 for key and Table 1 for strain details and protein accession numbers). Basic amino acids are highlighted in bold type. **(C)** Close-up of *S. solfataricus* MCM β9-β10 hairpin loop. **(D)** Location and nature of intended mutations in *Hfx. volcanii* MCM protein. Attempts were made to construct six paired alanine substitutions (bH1–6) and four short deletions (hD1–4). See text for details.

Structural analysis of archaeal MCM proteins identifies four β-hairpins in each monomer, three of which protrude, to a greater or lesser extent, into the central channel through which single- or double-stranded DNA is thought to pass (Slaymaker and Chen, 2012). One of these hairpins, the positively charged β9-β10 β-hairpin (also known as the NT-hairpin) is located in the N-terminal domain of the protein (see Figure 5A). Unlike the β7-β8 loop described above, the sequence of this part of the MCM protein is not well-conserved across evolution (Figure 5B). However, it appears that the positively charged nature of the hairpin is important for function: mutation of arginine 226 and lysine 228 in the *M. thermoautotrophicum* MCM abolishes that protein's ability to bind to DNA (Fletcher et al., 2003).

To probe the *in vivo* function of the β9-β10 β-hairpin loop in *Hfx. volcanii*, and in the absence of highly conserved amino acids presenting themselves as obvious targets for mutagenesis, we initially attempted to construct six mutants, *mcm-bH1*—*mcm-bH6*, in which adjacent amino acids were replaced with paired alanines (Figure 5D). All six mutants were recovered following PCR screening (and confirmed by sequencing, see Supplementary Figure S2), implying that the β9-β10 β-hairpin loop is readily mutable. None of six mutants exhibited obvious growth defects at 45°C (Figure 4A, middle panel).

We therefore extended this analysis by attempting to create strains carrying deletions of increasing size in the β9-β10 loop (mutants *mcm-hD1*—*mcm-hD4*, see Figure 5D). However, despite extensive screening, only two of the four could be isolated: *mcm-hD1* and *mcm-hD2*. Growth of these strains, like *mcm-bH1*—*mcm-bH6*, was indistinguishable from the parental wild-type H26 (Figure 4A, lower panel). That we were unable to isolate *mcm-rD3* and *mcm-rD4* strains strongly implies that these deletions either inactivate or significantly impair the function of the *Hfx. volcanii* MCM protein. We conclude from this analysis that while the precise sequence of the β9-β10 β-hairpin loop is not absolutely required for the function of the protein, the loop itself does have a crucial role.

### Mutagenesis of the zinc binding domain

**Figure 6:**
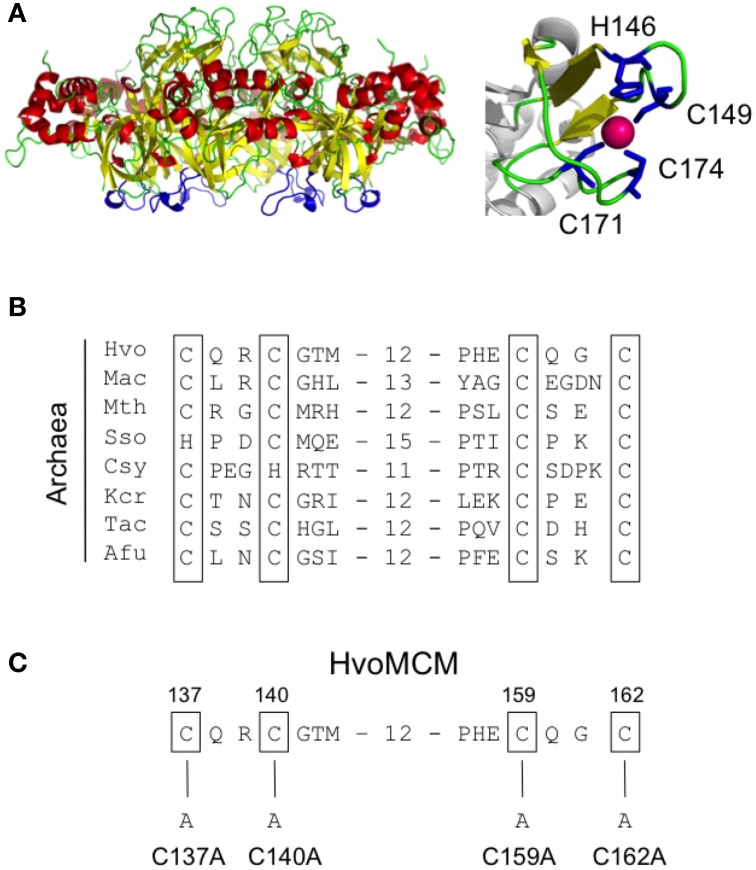
**Mutagenesis of the zinc binding domain. (A)** Structure of the *S. solfataricus* MCM N-terminal domain hexamer (PDB 2VL6) with the zinc binding domains highlighted in blue, alongside a close-up view showing the coordination of zinc by the four conserved cysteines. **(B)** Multiple sequence alignment of the zinc binding domain region from diverse archaeal species (see legend to Figure 3 for key and Table 1 for strain details and protein accession numbers). Conserved cysteines are shown boxed. **(C)** Location and nature of intended mutations in *Hfx. volcanii* MCM protein. Attempts were made to construct four cysteine-to-alanine point mutants. See text for details.

The N-terminal domain of the archaeal MCM proteins contains cysteine and histidine residues that fold into a zinc binding domain (Figure 6) (Slaymaker and Chen, 2012). A similar domain appears also to be present in eukaryotic MCM proteins but its precise function in either kingdom is unknown. We attempted to construct mutants in which each of the four cysteines was individually replaced with alanine (Figure 6C). However, none of these four mutants that we attempted to construct (mutants *mcm-C137A*, *mcm-C140A*, *mcm-C159A*, and *mcm-C162A*, see Figure 6C) could be isolated, implying that all four cysteines—and presumably zinc binding—is essential for MCM function *in vivo*. As a control, we tested whether it was possible to introduce a silent mutation into the vicinity of the cysteine 140 codon by changing the sequence GGGACG encoding glycine 141 and threonine 142 to an *Acc*65I restriction site, GGTACC (mutation *mcm-S1*). Sixty colonies were screened by *Acc*65I digestion of the PCR amplified *mcm* gene: 26 contained the Acc65I site (data not shown), indicating that the region of the gene encoding the zinc binding domain can be mutated in this manner.

### Sensitivity to mitomycin C

In total, we isolated 13 mutant strains carrying either single alanine substitutions, paired alanine substitutions and short sequence deletions in two conserved sequence elements, the β7-β8 and β9-β10 β-hairpin loops. None of these strains displayed obvious growth deficiencies: none of the strains grew slowly (Figure 4A) nor were any of the strains cold-sensitive or temperature-sensitive (data not shown). We also tested whether the strains might be sensitive to DNA damage induced by the DNA modifying agent mitomycin C (MMC). MMC forms three types of MMC-DNA adducts: monoadducts, intrastrand biadducts and interstrand crosslinks (Tomasz, 1995; Bargonetti et al., 2010). Of the 13 tested strains, one strain, *mcm-bH5*, showed increased sensitivity to MMC treatment (Figure 4B). This mutant has two adjacent residues in the β9-β10 β-hairpin loop, asparagine 234 and glutamate 235, replaced with alanine.

## Discussion

The MCM helicase is the key catalytic engine of DNA unwinding during chromosome replication in eukaryotes and most likely in archaea also. Three factors make the archaeal MCM proteins excellent models for their human counterparts. First, the relatively high level of sequence similarity between the eukaryotic and archaeal MCM proteins throughout the non-catalytic N-terminal and catalytic AAA+ domains. Second, the relative simplicity of the homohexameric archaeal MCM complexes compared to the heterohexameric eukaryotic complexes. Third, the relative ease with which certain archaeal MCM proteins can be purified in recombinant form, assayed for various activities and crystallized for structure determination. It is striking that although more than 10 years have passed since publication of the first partial crystal structure of an archaeal MCM (Fletcher et al., 2003), no high-resolution eukaryotic MCM structures have been solved. Until this occurs, the archaea will remain an important model allowing detailed dissection of MCM protein function.

Using multiple protein sequence alignments and crystal structures as a guide, a number of groups have identified regions of the MCM protein with potentially important roles in MCM function. These have then been mutated and the consequences for MCM activity determined by a variety of *in vitro* biochemical assays. The β7-β8 β-hairpin loop was first identified in this way, for example (Sakakibara et al., 2008; Barry et al., 2009). However, despite the growing number of similar studies in the literature, no attempt has been made to examine the *in vivo* consequences of such mutations.

Here we describe the results of probing the *in vivo* function of specific amino acids within an archaeal MCM protein using the haloarchaeon *Hfx. volcanii* as a model. *Hfx. volcanii* encodes a single MCM protein of 702 amino acids. This protein is part of the core group of haloarchaeal MCM proteins defined in a previous study (MacNeill, 2009) and expanded upon here. Each of 26 haloarchaeal species investigated encodes a single member of this group (Table 1, Figure 1). Ten species encode additional MCM family proteins classed as outliers. Given that a single core protein is found in all species examined and that 16 of the species encode only a single MCM, it is highly likely that these proteins act as replicative helicases. In support of this, the single MCM protein encoded by *Hbt. salinarum* NRC-1 (equivalent to the *Hbt. salinarum* R1 protein listed in Table 1 and labeled as number 4 in Figure 1) has previously been shown to be essential for cell survival (Berquist et al., 2007). While we have not attempted to delete the *Hfx. volcanii mcm* gene in its entirety, our inability to isolate certain mutant *mcm* alleles in this study strongly points to the *Hfx. volcanii mcm* being essential also.

The cellular functions of the outlying haloarchaeal MCM proteins are unknown. With the exception of the group of four C-terminally truncated MCM proteins highlighted in Figure 1 (proteins labeled 5B, 11C, 17B, and 19B), all the outliers possess intact Walker A, Walker B and arginine finger motifs, suggesting that they may be active as ATPase and/or DNA helicases. Interestingly, *T. kodakarensis* encodes three MCM proteins, all three of which have helicase activity, although only one is essential for cell viability and which therefore is the likely replicative helicase (Ishino et al., 2011; Pan et al., 2011).

In order to determine whether *Hfx. volcanii* presented a workable model for reverse genetic analysis of archaeal MCM function, we targeted three regions of the proteins for investigation. Initially, we focused on the β7-β8 β-hairpin loop located at the interface between the N-terminal and catalytic domains (Figure 3A) and previously identified as having a role in communicating conformational changes from the DNA binding N-terminal domain to the catalytic AAA+ domain (Sakakibara et al., 2008; Barry et al., 2009). The β7-β8 loop is well conserved across eukaryotic and archaeal evolution (Figure 3B). In this study, we constructed five strains (*mcm-rM1*, *mcm-rM3—mcm-rM6)* carrying single point mutations in the *Hfx. volcanii* MCM β7-β8 β-hairpin loop (Table 2, Figures 3, 4). The mutated residues included glutamine 186, which is conserved in both archaeal and eukaryotic MCM proteins (Figure 3B) but which can be replaced by alanine without significantly affecting cell growth, as well as three charged residues (glutamates 190 and 196, and arginine 193) and glutamine 199, all of which were also replaced by alanine without markedly affecting growth rates. In addition, none of the five β7-β8 loop mutants led to increased sensitivity to MMC exposure in spotting assays (Figure 4).

In contrast, we were unable to isolate mutant *mcm-rM2* encoding a protein in which conserved residue glutamate 187 was replaced by alanine, nor any of the four deletion alleles *mcm-rD1*—*mcm-rD4*. Replacement of glutamate 182 in the *M. thermoautotrophicum* MCM protein, the residue corresponding to glutamate 187 in *Hfx. volcanii*, with arginine led to marked reductions in ATPase and helicase activities *in vitro*, without affecting either ATP or DNA binding (Sakakibara et al., 2008), whereas mutation of conserved glutamine 181 (equivalent to glutamine 186 in *Hfx. volcanii* MCM, the residue mutated in *mcm-rM1*) to alanine had little impact. Clearly, conservation is no predictor of *in vivo* essentiality.

We turned next to the β7-β8 β-hairpin loop, also known as the NT-hairpin. This loop extends into the central channel of the MCM hexamer and may have a role in tracking DNA through the channel based on the fact that mutating two positively charged residues at the tip of the loop in the *M. thermoautotrophicum* MCM protein abolishes DNA binding *in vitro* (Fletcher et al., 2003). In *H. volcanii*, only a single basic residue is present in the β9-β10 loop region, arginine 236. This is likely to lie at or near the tip of the β-hairpin (Figures 5B,C). By comparison with *M. thermoautotrophicum*, it would be reasonable to predict that this amino acid would be essential for N-terminal domain DNA binding and thus for MCM function *in vivo*. In the absence of more widespread sequence conservation, we chose to probe the *in vivo* function of the β7-β8 β-hairpin loop initially by attempting to construct a series of six mutants in which adjacent amino acids were replaced with pairs of alanines (Figure 5D). All six mutant strains (Table 2, Figures 3, 5) were viable, including *mcm-bH6* in which arginine 236 was replaced with alanine. Next, we attempted to construct four different β9-β10 loop deletion mutants but only two were viable: *mcm-hD1* and *mcm-hD2* (Table 2, Figures 3, 5). The former removes amino acids 231 and 232, and the latter amino acids 230–233. In contrast, strains carrying two larger deletions, *mcm-hD3* and *mcm-hD4*, removing amino acids 229–234 and 228–235, respectively, could not be isolated. Thus, these results clearly demonstrate that while no individual amino acid within the β9-β10 hairpin loop is essential for *Hfx. volcanii* MCM function *in vivo*, the loop does have a key role to play.

In addition to mutagenizing the β7-β8 and β9-β10 loops, we also attempted to individually replace with alanine each of the four cysteine residues that make up the zinc binding domain (Figure 6). However, we were unable to recover any of four desired mutants, *mcm-C137A*, *mcm-C140A*, *mcm-C159A*, or *mcm-C162A*, and conclude that the zinc binding domain is therefore likely to have an essential function *in vivo*. Consistent with this, replacing the equivalent of *Hfx. volcanii* MCM cysteine 162 with serine produces an *M. thermoautotrophicum* MCM protein with impaired ATPase and single-stranded DNA binding activities and no helicase activity (Poplawski et al., 2001).

Finally, the mutant *Hfx. volcanii* strains generated in this study were tested for increased sensitivity to the DNA modifying drug mitomycin C (MMC). MMC is a potent DNA interstrand crosslinker and is widely used as replication fork blocking agent, as replication cannot continue past such crosslinks. We tested all 13 viable *mcm* alleles for MMC sensitivity by spotting serially diluted cultures onto medium containing increasing concentrations of MMC and found one strain, *mcm-bH5*, that displayed significantly enhanced sensitivity compared with the parental wild-type H26 (Figure 4B). On medium lacking MMC, growth of *mcm-bH5*, as with the other viable β9-β10 loop mutants, is indistinguishable from wild-type (Figure 4A). The *mcm-bH5* protein carries a double substitution in β9-β10 loop with asparagine 234 and glutamate 235 being replaced with a pair of significantly less bulky alanines. In the absence of a crystal structure of the *Hfx. volcanii* MCM protein, it is difficult to predict whether the impact of the *mcm-bH5* mutations is confined to the β9-β10 loop alone or whether these amino acid changes will have a wider impact on N-terminal domain structure. It is also difficult to envisage how the *mcm-bH5* mutations would cause cells to become supersensitive to MMC, particularly as interpreting the effect of MMC on cells is complicated by the different modes of action of this compound. MMC forms at least four different types of DNA adduct: two species of MMC-mono-dG-adduct, intra-strand dG-MMC-dG biadducts and inter-strand dG-MMC-dG crosslinks, with the latter being assumed to be the replication fork blocking lesion (Tomasz, 1995; Bargonetti et al., 2010). Given the key role of the MCM helicase at the fork it is tempting to speculate that the supersensitivity of *mcm-bH5* is a result of difficulties that arise when the mutant helicase encounters an inter-strand MMC crosslink. However, confirmation of this clearly requires additional experimentation.

In conclusion, the results presented here demonstrate the feasibility of using *Hfx. volcanii* as a model system for reverse genetic analysis of archaeal MCM protein function, provide important confirmation of the *in vivo* importance of conserved structural features identified by previous bioinformatic, biochemical and structural studies, and offer the prospect of more extensive mutational analysis, not only of MCM but of other key replication factors, in the future.

## Author contributions

Tatjana P. Kristensen, Reeja Maria Cherian, and Fiona C. Gray performed the bulk of the experimental work. Stuart A. MacNeill designed the study, carried out some of the experimental work and wrote the manuscript.

### Conflict of interest statement

The authors declare that the research was conducted in the absence of any commercial or financial relationships that could be construed as a potential conflict of interest.

## Supplementary material

Supplementary Figure S1 **Sequence analysis of β7-β8 β-hairpin mutant strains**. Codons mutated to encode alanine are boxed.

Supplementary Figure S2 **Sequence analysis of β9-β10 β-hairpin mutant strains**. Codons mutated to encode alanine pairs (mutants bH1–6) are boxed. Vertical lines in hD1 and hD2 indicate location of deleted sequences.
