# Supplementary material for: The haloarchaeal MCM proteins: bioinformatic analysis and targeted mutagenesis of the β7-β8 and β9-β10 hairpin loops and conserved zinc binding domain cysteines
Source: Front Microbiol. 2014 Mar 26;5:123. doi: 10.3389/fmicb.2014.00123 (PMC3972481; doi:10.3389/fmicb.2014.00123)

Supplementary Figure S1

Sequence analysis of β7-β8 β-hairpin mutant strains. Codons mutated to encode alanine are boxed.

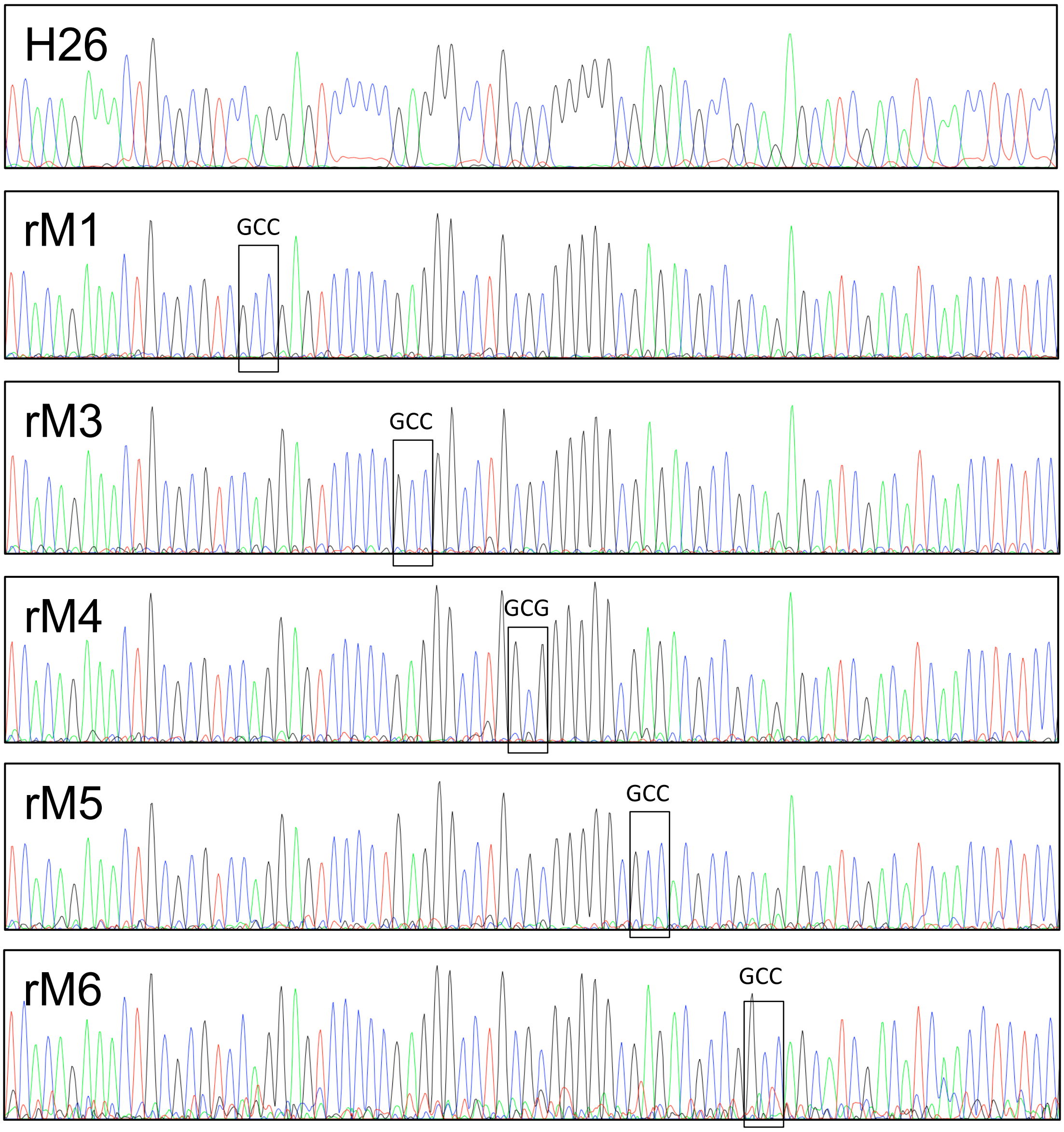

Supplementary Figure S2

Sequence analysis of β9-β10 β-hairpin mutant strains. Codons mutated to encode alanine pairs (mutants bH1-bH6) are boxed. Vertical lines in hD1 and hD2 indicate location of deleted sequences.

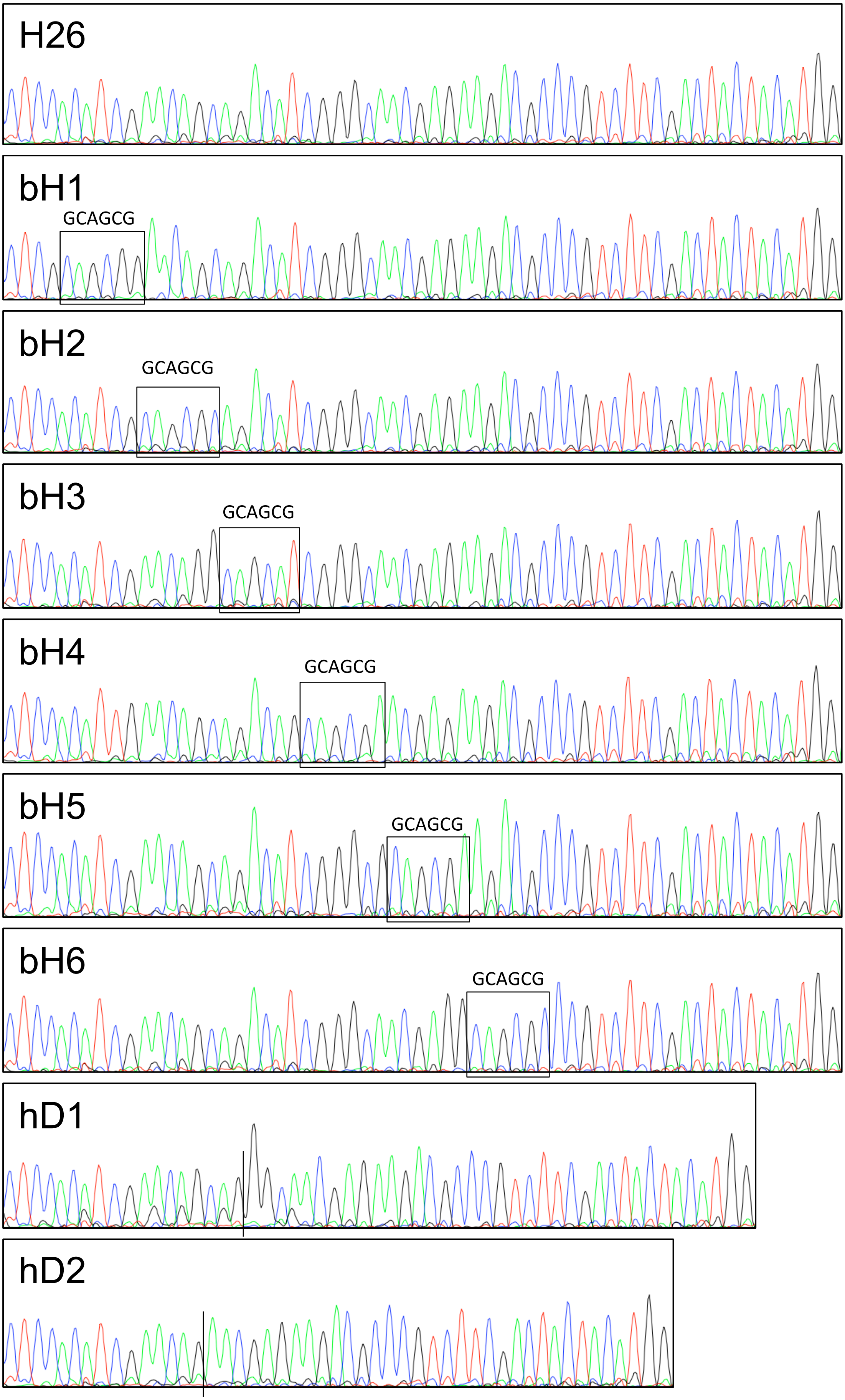

Supplement: Supplementary Figure S1 — Sequence analysis of β7-β8 β-hairpin mutant strains. Codons mutated to encode alanine are boxed. [file DataSheet1.PDF]
